# The moral case for sign language education

**DOI:** 10.1007/s40592-019-00101-0

**Published:** 2019-11-23

**Authors:** Hilary Bowman-Smart, Christopher Gyngell, Angela Morgan, Julian Savulescu

**Affiliations:** 1 Biomedical Ethics Research Group, Murdoch Children’s Research Institute, 50 Flemington Rd, Parkville, VIC 3052 Australia; 2 Department of Paediatrics, University of Melbourne, Parkville, VIC 3010 Australia; 3 Speech and Language Group, Murdoch Children’s Research Institute, 50 Flemington Rd, Parkville, VIC 3052 Australia; 4 Department of Audiology and Speech Pathology, University of Melbourne, Parkville, VIC 3010 Australia; 5 Uehiro Centre for Practical Ethics, University of Oxford, St Ebbes St, Oxford, OX1 1PT UK

**Keywords:** Sign language, Auslan, BSL, Deaf culture, Education, Language education

## Abstract

Here, a moral case is presented as to why sign languages such as Auslan should be made compulsory in general school curricula. Firstly, there are significant benefits that accrue to individuals from learning sign language. Secondly, sign language education is a matter of justice; the normalisation of sign language education and use would particularly benefit marginalised groups, such as those living with a communication disability. Finally, the integration of sign languages into the curricula would enable the flourishing of Deaf culture and go some way to resolving the tensions that have arisen from the promotion of oralist education facilitated by technologies such as cochlear implants. There are important reasons to further pursue policy proposals regarding the prioritisation of sign language in school curricula.

## Introduction

Learning another language is a life goal for many. We generally think that doing so is a form of self-improvement. We teach languages in our schools. Many people go out of their way to ensure that their child becomes bi- or multilingual. However, it is important to ask exactly why we choose certain languages above others, and which languages we should teach our children. The answer depends on assessing not just what is good for the individual child, but also what makes society better.

Languages taught in schools in English-speaking countries are often European or Asian languages. In Australia, Japanese, Italian, French, Indonesian, German and Chinese constitute 93% of enrolment numbers (Orton 2016). In both the United States (US) and the United Kingdom (UK), Spanish, French and German dominate (American Council on the Teaching of Foreign Languages 2011; Long and Bolton 2016). One set of languages that receives comparatively little attention are sign languages such as Australian Sign Language (Auslan), British Sign Language (BSL) and American Sign Language (ASL).

In December 2016 the first Australian national curriculum for Auslan was launched, as a result of much lobbying from the deaf (or Deaf) community (Dalzell 2016). This means there is a standard text for teaching Auslan that can be implemented around the country. Despite this, Auslan is still only taught in 4% of all Victorian public schools (Hore 2017). A recent push has occurred in the UK for BSL to be made available as a GCSE subject, with one child mounting a legal challenge in 2018 (Busby 2018). A 2017 petition to the government calling for BSL to be integrated into the national curriculum received over 35,000 signatures (“Make British Sign Language part of the National Curriculum” 2018). A 2017 survey by the National Deaf Children’s Society found that 92% of young people (both deaf and hearing) thought BSL should be taught in schools (National Deaf Children’s Society 2017). In the US, the provision of ASL in secondary schools is increasing, although it remains a very small minority of foreign language enrolments; teachers generally rely on a number of commercially-prepared curricula (Rosen 2010).

Here, we argue that sign languages should be compulsorily integrated into the school curriculum, whether primary, secondary, or both. This would make sign language education accessible to both hearing and deaf or hard-of-hearing students. We will focus on English-speaking countries as examples, particularly Australia (with Auslan) and the UK (with BSL). In these two countries in particular, sign language education has been the matter of recent public debate. We do not propose a specific educational policy, but rather a moral case as to why sign languages should be prioritised in any approach to developing a school curriculum.

Although the strong version of our claim is that sign language should be made compulsory, we accept that there may be some situations and contexts where this may not be appropriate or possible. In these exceptions, we still argue that sign language education should at least be made accessible, prioritised, and/or incentivised.

Teaching a second language has many cognitive and social benefits. Teaching sign language, specifically, has further benefits. Firstly, learning sign language would benefit individual students, as it would improve each student’s overall communication skills and provide additional cognitive advantages that come from being bimodally bilingual. Secondly and critically, widespread knowledge of sign language would benefit numerous groups who are already disadvantaged, such as those with a communication disability, particularly those who are congenitally deaf or hard-of-hearing. These individuals are at risk for social isolation, stigmatisation, loss of independence, poorer literacy and academic outcomes, underemployment, and overrepresentation in the juvenile justice system (Bryan et al. 2010; Health Workforce Australia 2014; Law et al. 2009; Schoon et al. 2010; Snow and Powell 2007). This makes sign language education a question of justice. Thirdly, teaching sign language in schools will go some way to resolving the tension around new technologies and the erasure of Deaf culture.

We set out our case as follows. In Sect. 2, we outline the benefits that learning sign language bestows on individuals. In Sect. 3, we argue that considerations of justice favour prioritising the teaching of sign language over other second languages. In Sect. 4, we discuss issues regarding identity and deaf culture. We conclude by endorsing the general principle that in a default curriculum, students should learn sign language. At the very least, sign languages should be much more widely taught than they are now, so that they are among the most widely taught languages.

## Benefits to the individual

Learning a second language has a number of demonstrated benefits to the individual. It can foster analytic thinking (Jiang et al. 2016), enhance multitasking (Poarch and Bialystok 2015), and improve social cognition and executive control (Bialystok and Craik 2010; Carlson and Meltzoff 2008; Colzato et al. 2008; Cox et al. 2016; Hilchey and Klein 2011) among a number of other cognitive benefits. These benefits are most evident when the second language is supported with strong bilingual education rather than only speaking the language at home (Lauchlan et al. 2012). Numerous studies have indicated that bilingualism serves as protection against cognitive decline in older age, delaying the onset of dementia by 4 to 5 years (Alladi et al. 2013; Perani et al. 2017; Woumans et al. 2015). With this level of protection against age-related disease, language education could even be argued to be a kind of public health measure. Additionally, there is the simple positive aspect of being able to communicate directly with a larger number of people than one otherwise would be able to. This also means the opportunity to engage with other cultures to a deeper level.

Learning a sign language provides additional benefits, as not only does it make a person bilingual, but also bimodal. It provides several cognitive gains: it improves the use of co-gesture in speech (Casey et al. 2012), improves the ability to identify facial expressions (Bettger et al. 1997), enhances vocabulary development and literacy in young children (Daniels 1994, 2004; Moses et al. 2015), and improves spatial cognition such as mental rotation (Emmorey et al. 1993, 1998; Romero Lauro et al. 2014; Talbot and Haude 1993). Bimodal bilinguals can co-activate both languages during spoken comprehension (Shook and Marian 2012) and there is no cost to simultaneous speech and sign (Emmorey et al. 2016). Uniquely, sign language allows for simultaneous communication in two modalities; this is not possible with two oral languages.

There are additional social benefits to learning sign language. For example, it allows people to communicate in very noisy environments (such as a crowded bar or factory) or in an unobtrusive fashion where noise may not be allowed or may be distracting (such as the classroom). It facilitates effective communication with members of the deaf community who do not communicate orally, without the need of an interpreter or assistive device (including pen and paper). This can have advantages in both the personal and professional realm (for example, by making a business more accessible to deaf or hard-of-hearing people, thereby potentially increasing profit).

Gestures and visual communication are already an integral part of communication, with co-gestures representing an important visual modality that accompanies verbal output (Perniss et al. 2015). Sign language further provides another modality beyond the verbal to express oneself. A large part of communication is non-verbal, and the use of sign language integrates, formalises and expresses this non-verbal communication in an effective way. The strong link between sign language and emotional expression (Elliott and Jacobs 2013) may prove to be a positive outlet for some.

Learning sign language will also provide additional benefits to those who may *become* deaf. Hearing loss is associated with age (Oh et al. 2014). Australia’s population is ageing, and the proportion of Australians who are 65 or older is expected to continue to grow, projected to reach a quarter of the population by the end of this century (Australian Institute of Health and Welfare 2018). The situation is similar in the UK (Office for National Statistics 2017). Therefore, the number of people in these countries who are deaf or hard-of-hearing is likely to increase. Admittedly, technological and medical progress may prevent this, but this is not guaranteed. Loss of hearing due to age has been associated with impacts on quality of life, social relationships, and cognitive function (Fortunato et al. 2016). Learning sign language prior to the advent of hearing loss could ameliorate these impacts and make this transition less distressing. Learning another language such as German does not necessarily provide a benefit in the same way. For example, if you failed to learn German before you moved to Germany, you would be in a difficult position. However, apart from taking language lessons, you could also turn to a translator to translate German into your primary language. If you become hard of hearing when you rely on oral communication, you have lost your full capacity to communicate in your primary language. Without knowledge of sign language, a translator will provide no additional benefit to you. You have not just moved to another country where people do not understand you; there is no chance of going home. This is a reason that teaching sign language specifically confers a benefit to the individual over the teaching of other second languages. Over time, as younger generations transition, it would allow effective communication with the elderly as they become hard of hearing, without requiring hearing assistive technology.

In sum, the learning of sign language will benefit individuals by promoting a bimodal form of communication that can facilitate expressive communication. These benefits will be particularly significant for those who are, or will become, deaf. It should be noted, however, that the degree to which individuals (as well as society in general) will benefit depends on the degree to which students successfully acquire sign language in school, and the extent to which they will retain it throughout their lifetime. This is hard to predict prospectively. We will simply note even if people only acquired a small amount of sign language, this could have significant benefits in terms of the social acceptance of deaf culture. Furthermore, learning a little sign language at school would provide a platform on which to learn further sign language when needed (for example, if they become deaf).

## Justice

The Roman lawyer Cicero gives one of the earliest definitions of justice as “the virtue which assigns to each his due” (Cicero 1933). This broad definition still captures the core concerns of justice today. While justice encompasses many elements of ethics and law, it fundamentally represents a concern for giving people what they are ‘due’. Educational resources (such as a teacher’s time, a school’s budget) are limited. This raises the question of how we ought to allocate these resources in a way that assigns each their due.

There are several different theories of distributive justice which give different answers to the question of how we should distribute a limited resource. However, the most widely accepted theories are versions of ‘prioritarianism’ (Parfit 1997), which is the view that, other things being equal, benefits matter more, the more worse off their recipients.^1^ One version of prioritarianism is John Rawls’ theory of justice, which includes the difference principle (Rawls 1999). It holds that differences between the best-off and the worst-off are only permissible if they raise the absolute standing of the worst off. On this view, we should distribute educational resources so that the worst off in society are as well off as they possibly can be.

Teaching sign language will benefit groups in society who are significantly marginalised. In many contexts, there will be strong reasons of justice to prioritise teaching sign language over other languages, as outlined further here.

Being able to communicate is an essential component of being able to participate fully in society. For this reason, it is more important to teach children a language that will allow them to communicate with those whose capacities for communication and engagement with society are limited by a language barrier. This is not necessarily the case with all second languages currently being taught, such as some European languages of wealthy countries where migrants are likely to be highly educated and already speak English. Therefore, linguistic minorities that are less likely or able to speak English have a greater claim to their language being represented on the educational curriculum than those who can speak English.

With this line of argument, it is also important to establish the deaf or hard-of-hearing not only as a linguistic minority, but also as a marginalised minority who are worse-off in a way that is directly related to language. It is more difficult for people who are deaf to communicate with other members of society and go about their daily lives with the ease of those who are not deaf can do. Although many people do not recognise deafness as a disability in itself (Bauman et al. 2014), the social implications of not being able to communicate in the same way as the majority of society are clear (regardless of how we conceptualise these barriers). It is, for example, more difficult to order a coffee or open a bank account if there is nobody who can communicate with you simply and effectively by non-oral means—that is, through languages such as Auslan. These are relatively trivial tasks, but it is also evident in more serious and important moments in life, such as being unable to communicate with medical staff during the birth of your child (Browne 2016). There is evidence of discrimination against the deaf in both Australia and the UK. Those who are deaf have poorer employment outcomes (Hill et al. 2017; Willoughby 2011; Winn 2007); as of 2015 in Australia, people with a communication disability such as deafness have a labour participation rate of only 37.5% (Australian Bureau of Statistics 2017a). Deaf people have increased difficulty with accessing primary healthcare services (Kuenburg et al. 2016), and in the UK, deaf mental healthcare service users stay in hospital twice as long as hearing patients (Baines et al. 2010). Deaf people have increased barriers accessing the criminal justice system in the UK (Elder and Schwartz 2018) and are not able to serve as jurors in Australia (Napier and McEwin 2015). Parents of deaf children have had to resort to the courts to ensure that their children receive education that is accessible to them (Busby 2018; Komesaroff 2004). Although some of these problems are systemic and institutional, if the number of people who were able to communicate in sign languages were to increase, even if that level of communication is not particularly strong or skilled, this will go some way to ameliorating the difficulties deaf people face as they go about their daily life. It will also normalise the use of sign languages in various contexts and could provide a societal background where discrimination against the deaf is less accepted.

Deafness, as noted above, also intersects with other marginalised groups such as the elderly. Forms of sign language can also be useful for students with intellectual and developmental disabilities, such as autism spectrum disorder, Down syndrome (Toth 2009), or the plethora of genetic syndromes identified in this genomic era. Emphasising alternate modalities of language may help in making these communication methods more accessible and/or normalised. Modified forms of sign language, such as Key Word Sign or Makaton, have proved highly valuable for people with intellectual disabilities (Beecher and Childre 2012; Meuris et al. 2015; van der Meer et al. 2012). Teaching sign language may benefit individuals with, for example, autism, either directly or indirectly by making communication with their friends, family members and support staff easier. Varieties of sign languages can form a part of or a more natural alternative to augmented communication devices, and increased knowledge may be helpful for those who require access to alternative or augmented communication. However, it is important to recognise here that in this context we are not referring specifically to sign languages such as Auslan or BSL. Auslan and BSL are not in any way ‘easier’ or less complex than spoken languages. Rather, we argue that the broader implementation, integration and normalisation of bimodality may foster a more conducive environment for those with other forms of communication disabilities. Having some knowledge of sign language may make it more accessible for people to use other forms of signed language to facilitate communication.

There is an additional key difference that makes it more just to learn a sign language than the languages of other marginalised linguistic minorities. Simply put, it is possible for someone who speaks French to learn how to speak English. Although there may be barriers for many people to learn another language (including, for example, access to educational resources and/or time to learn), and this should certainly be taken into consideration, second language learning is still generally possible. It is very difficult or impossible for someone who is profoundly deaf to communicate verbally in English or comprehend spoken English, particularly if they have not learnt to do so at a young age or prior to hearing loss. Communication in writing is not sufficient compensation. The language barrier is one of function and cannot be overcome by the deaf party learning another language. Thus, it is the onus of those who speak English to learn the most effective language with which to communicate—that is, sign language.

The benefits to deaf people do not just extend to being able to access more goods and services directly. Even with widespread integration of sign language into a curriculum, there will remain many hearing people who require an interpreter when communicating with deaf people. Deaf people have a right to communicate through an interpreter, particularly when it comes to vital services such as medical care. There are a limited number of sign language interpreters, and more are needed. In Australia, with the recent introduction of the National Disability Insurance Scheme (NDIS), the demand has increased and it is a matter of justice for this demand to be met (Campbell 2018). Three out of five children under 12 living with a communication disability, including deafness, have unmet needs for formal communication assistance (Australian Bureau of Statistics 2017a). Similarly, a 2017 government review found that there is a significant shortage of interpreters in the UK, with only 908 registered sign language interpreters in the entire UK (Department of Work & Pensions 2017). Integrating sign language into school curriculums will increase the exposure of young people to sign language, and may influence the number of those who choose to become interpreters. There is also the risk that speech pathology services may deteriorate in quality due to the increase in demand caused by the NDIS (Health Workforce Australia 2014), and so the ability to use a non-oral language to communicate may become even more important.

It is important that the training and work of skilled and certified sign language interpreters would remain essential, even if there were more widespread knowledge of sign language. Some knowledge of a language would not be sufficient to provide translation services in an ad-hoc fashion in the context of medical care, education, or public events. A skilled interpreter would absolutely be required in many situations. There is the risk that some may overestimate their ability to communicate in sign language and thus counterproductively impair effective communication. However, in small daily tasks where an interpreter is unlikely to be resourced, some knowledge of a language—such as numbers, and common words—would facilitate effective communication.

A greater emphasis on learning sign language at schools can also serve to rectify historical injustices. The Milan conference of 1880 solidified the teaching of the oralist tradition and greatly discouraged the use of sign languages in deaf education (Moores 2010). This has had profound impacts on deaf pedagogy and the growth and development of sign languages. For a long time, sign languages were not seen as legitimate languages. Although the teaching of sign language in schools cannot rectify the harms already done to those who were unable to fully master, learn, or communicate in the most appropriate language for their needs, it can go some way to legitimising sign language as a valuable form of communication that should be encouraged.

Another justice-based reason to prioritise teaching sign language over other languages is that it would enable countries such as Australia and the UK to fulfil their obligations under The United Nations Convention on the Rights of Persons with Disabilities (CRPD), which was ratified in 2009 by both Australia and the UK (Australian Law Reform Commission n.d.; Fraser Butlin 2011). By ratifying the CRPD, these governments imposed on themselves several obligations in relation to sign language including:

Facilitating the learning of sign language and the promotion of the linguistic identity of the deaf community;

Taking appropriate measures to employ teachers, including teachers with disabilities, who are qualified in sign language and/or Braille, and to train professionals and staff who work at all levels of education.

Given the lack of easy access to sign language education, there is an imperative on these governments to undertake more drastic means to increase the uptake of Auslan or BSL. Making sign languages compulsory in schools would be the most effective way to discharge their obligations in relation to the CRPD.

Amongst all this, there is the question as to whether the teaching of sign language will come at a cost to the individual. If it does, then this must be weighed against the benefits to others who are currently worse off or marginalised (i.e., the deaf). This cost to the individual student may be the provision of sign language education at the expense of another language that it is more in the student’s interests to learn, and that it may reduce the frequency of other forms of bilingualism on a population level. If this cost is significant (i.e. it affects many students), then this is reason to reconsider our contention. However, we do not believe that a policy of compulsory sign language education will make a large number of students or society worse off, at least in Australia and the United Kingdom.

Firstly, as of the 2016 Census, 21% of Australians speak a language other than English at home (Australian Bureau of Statistics 2017b). However, less than 10% of students learn language to a Year 12 level (and this includes those who are already native language speakers) (Mayfield 2017). This suggests that most of those who speak a second language do not learn it at school. In addition, schools frequently offer multiple languages, so that students can continue to pursue learning several languages. We accept that in the event that a school only has the resources to teach one language, there may be considerations for an exception to making sign language education available if there is another language with a greater claim to be prioritised in a particular context. Depending on regional context, there may be a case for another language to be prioritised along with, or instead of, sign language. For example, if there is an area in an English-speaking country with a high number of unilingual Spanish speakers, and resources or individual capacity are not available to facilitate learning English (including resources such as a person’s time or capacity), then Spanish has a strong case to be prioritised alongside (or instead of) sign language. Additionally, there might be reasons to promote the teaching of Indigenous languages over sign language in certain areas, to promote the continued survival of particular cultures.

These reasons stem from the same considerations of justice we have outlined earlier. However, we do not believe that exceptions such as these need to be widespread. This is because when we are addressing prioritisation of sign language education, the cost should be considered within the context of the whole curriculum rather than only the languages curriculum. We do not believe that generally, making sign language a necessary part of the curriculum need be an either/or proposition; there will be ways to implement sign language education without seriously impacting the provision of other second languages. For example, for schools with extremely limited resources or very low enrolments, an external sign language educational course could be established within the overall public school system, and students could be incentivised to attend.

Secondly, sign language is unique in a certain respect; most deaf children are born to hearing parents (Mitchell and Karchmer 2004), and so parents will often learn sign language (if they do at all) alongside the child whose native language it is likely to be. Children who are deaf do not have the opportunity to learn a language at home in the same way as many second language speakers. Therefore, the school is a key nexus for the spread of languages such as Auslan or BSL. There is much to suggest that language education in general is in serious need of investment,but there is also a strong argument that any attempts to overhaul or prioritise language education curriculums should focus on sign language(s). Even basic communication skills in sign language, rather than fluency, may have an important impact on society.

## Identity, Deaf culture, and language

There is significant tension between those who view deafness as something to be ‘fixed’, and those who view it as the basis for a rich cultural tradition (capital-D ‘Deaf’). This tension is exemplified in the debate around cochlear implants. Cochlear implants enable children with hearing loss to hear, with varying degrees of efficacy. It is generally encouraged to have cochlear implants implanted in children while they are very young, due to the sensitive period and neural plasticity that impacts their acquisition of oral communication (Tomblin et al. 2005). Implanting very young children with cochlear implants is done so that they grow up accustomed to the sensory input provided and more adequately adjust to oral communication methods.

However, some members of the Deaf community do not view cochlear implants as a positive development for deaf children. Rather, they view the advent of cochlear implants as facilitating a form of cultural erasure or ethnocide (Sparrow 2010). Deaf children undergoing cochlear implant surgery are thereafter generally raised in the ‘oralist’ tradition, where a strong emphasis is made on acquiring and practicing the skill of oral communication. This is at odds with a tradition more in line with the cultural model, which places an emphasis on sign language as a means of communication. Parents of children with cochlear implants have been discouraged by practitioners from signing with their children, with sign language viewed as a kind of ‘crutch’ that discourages effective oral communication (Humphries et al. 2017). Deaf children raised in an oralist tradition, with its strong emphasis on oral communication, are thus likely to learn sign language later in life, if they do at all. This may impact on their communication skills, their sense of identity, and their capacity to sign. Most deaf children are born to hearing parents (Mitchell and Karchmer 2004), and it is likely that hearing parents in general would wish their child to share their mode of communication—that is, oral language. This means that they may prioritise an oralist approach to what may be the detriment for the child.

Outcomes from cochlear implants vary greatly depending on the degree and nature of hearing loss (Cosetti and Waltzman 2012; Fontenot et al. 2018) and timing of implant (Dettman et al. 2016), and many children with cochlear implants do not attain the same level of spoken language outcomes as their non-deaf peers (Geers et al. 2009). Therefore, it would seem to remain beneficial on an individual level for children with cochlear implants to learn sign language. There is also, again, the level of group benefit. If there are fewer deaf people or people who view themselves as Deaf, the concern is that Deaf culture will lose many (potential) members. This sort of decrease in numbers of a cultural group is, naturally, generally seen as a negative by members of that culture who value its continued existence. Therefore, it would similarly be beneficial to a specific group of people (the culturally Deaf) that sign language be normalised and more widely taught and accessible, particularly to those who may otherwise have been discouraged from using it (deaf or hard-of-hearing people raised in an oralist tradition).

Much could be written on this source of disagreement between medical and cultural or social models of disability. However, this will not be explored at length here. It is sufficient to recognise that the Deaf community has a strong claim that their culture and practices are threatened by an emphasis on oral communication that is facilitated by the increased use of cochlear implants. However, there is concurrently a strong claim that children who are born deaf have the right to have access to the faculty of hearing if it is possible for them to do so (Byrd et al. 2011). It has been stated that cochlear implants provide the child with more of an ‘open future’ (Nunes 2001). Although the choice has been made by the parents to provide the deaf child with a degree of hearing, the child can later exercise that choice to reject the implant and the hearing abilities it provides. However, the reverse is not as true, as the older a child is when they receive a cochlear implant the less likely they are to effectively acquire oral communication (Boons et al. 2012). Therefore, providing young children with a cochlear implant may provide them with an increased range of options when making their life plans, if it is effective. Many culturally Deaf parents are now choosing cochlear implants for their children and raising them in a bimodal bilingual tradition (Mitchiner 2015).

It is important here not to assume that a deaf child would automatically be in favour of the use of a cochlear implant. Although teenagers with cochlear implants may generally view them positively (Wheeler et al. 2007), there are a number of cases where a cochlear implant may be rejected. This can be because the hearing facilitated by the cochlear implant is so poor as to be more of a hindrance than a help, dislike or pain associated with the sensation of hearing provided by the implant, difficulties with the extensive speech therapy generally required after cochlear implant surgery, or a rejection of the oralist tradition emblematised by the cochlear implant and concomitantly, an embracement of the Deaf identity (Watson and Gregory 2005). There are good reasons for the latter; involvement with the Deaf community has a positive impact on the mental health of deaf people (Fellinger et al. 2012). These may be valid and sensible reasons for an autonomous agent to reject the use of the cochlear implant in favour of their natural state of deafness.

However, while the decision to choose even a modicum of hearing over complete deafness is seen as the ‘obvious’ choice by hearing members of society, the converse choice to embrace deafness or Deafness is less understood and not necessarily seen as a reasonable choice to be supported. It is difficult to see how this choice between ‘hearing’ and ‘Deafness’ can be made in an autonomous fashion if the alternative option to oral culture—Deafness—is not sufficiently supported or validated by society. If a deaf person has not learnt sign language, how can it reasonably be said that they can make an autonomous and informed choice to embrace a Deaf identity with the ease that would have been provided to them if they had been raised in a manualist tradition? The debate may continue regarding the education of deaf children in their early years (a harm reduction approach would advocate for not depriving young deaf children of sign language regardless of cochlear implant status (Humphries et al. 2012)), but at least if all children learn sign language in school, this will allow children full access to both worlds and facilitate fully autonomous choice later in life. All children who have difficulties with hearing will be able to make an informed and autonomous choice about whether they identify as deaf, or Deaf, even if they have been raised with a focus on oralism, because access to a key part of Deaf culture—the language—will be normalised and made accessible to them by default. Importantly, it will also encourage a wider intercultural understanding, and this will reduce some of the difficulties associated with embracing Deafness, decreasing some of the pressures that may compel someone to make the alternative choice when they would prefer not to.

In addition to the direct benefits to the deaf child and family gained through significantly increased availability of sign language, there will also be broader cultural benefits to the Deaf community. If the number of people who have familiarity with sign language greatly increases, there will be advantages beyond the direct facilitation of communication. Even if students at school only learn rudimentary levels of sign language, the availability of and increased familiarity with sign language may have a positive impact on the wellbeing of the members of the Deaf community. This would be because increased availability of sign language could make Deaf people feel more included and welcomed in society. It would also facilitate societal familiarity with Deaf culture and validate deaf needs amongst hearing peers. If children lack familiarity with deafness and the needs of deaf people, they are more likely to view deafness negatively and be less likely to accept deaf peers (Batten et al. 2013). Therefore, even if sign language education does not produce widespread fluency, increased familiarity with elements of Deaf culture, such as sign language, are likely to have a positive impact on the Deaf community.

## Conclusion

There are a number of reasons why there is a strong moral claim for sign language to be compulsory, or at least highly prioritised, in the school curriculum. It would benefit individuals, and it would also benefit groups. The benefit to the deaf and/or culturally Deaf is a matter of justice, as the cost to the individual and other groups would be slight. Indeed, learning a sign language may provide hearing children with unique benefits. People living with a communication disability are also significantly marginalised, economically disadvantaged and have unmet needs for assistance; widespread knowledge of sign languages would ameliorate some of these associated negative impacts. It would also enable deaf or hard-of-hearing people to make an autonomous choice between hearing culture and Deaf culture, or embrace both.

As noted previously, this argument presents a moral perspective rather than a specific policy proposal. Here, we have outlined the ethical reasons why such policy proposals should be pursued and prioritised. In order to translate this into more concrete plans of action, extensive consultation would be required with the Deaf community, as well as service providers, teachers, and other education professionals.

It is a responsibility of society to create an *environment* that is most conducive to the welfare of everyone, including deaf people. Part of this process would be ensuring that as many people as possible can communicate in the most appropriate languages for the needs of this community, which are sign languages. The most effective way to ensure that as many people as possible would communicate in a sign language would be to integrate sign languages into the school curriculum.

## Footnotes

1 Two other view of distributive justice are egalitarianism—which aims for a distribution in which all are equal; and utilitarianism, in which resources should be distributed to provide the greatest benefits to the greatest number. Both are subject to serious objections as theories of justice. For example see Crisp, Roger. 2003. ‘Equality, Priority, and Compassion’ *Ethics* vol. 113, Issue 4: 745–763 and Dworkin, Ronald, 2000, *Sovereign Virtue: the theory and practice of equality*, Cambridge, MA: Harvard University Press.
